# Enhanced Reduction of Few-Layer Graphene Oxide via Supercritical Water Gasification of Glycerol

**DOI:** 10.3390/nano7120447

**Published:** 2017-12-14

**Authors:** Daniel Torres, Pedro Arcelus-Arrillaga, Marcos Millan, José Luis Pinilla, Isabel Suelves

**Affiliations:** 1 Instituto de Carboquímica, CSIC, Miguel Luesma Castán 4, 50018 Zaragoza, Spain; dtorres@icb.csic.es (D.T.); isuelves@icb.csic.es (I.S.); 2 Department of Chemical Engineering, Imperial College London, London SW7 2AZ, UK; pedro.arcelus-arrillaga09@imperial.ac.uk (P.A.-A.); marcos.millan@imperial.ac.uk (M.M.)

**Keywords:** few-layer graphene oxide, reduced graphene oxide, supercritical water, glycerol gasification, hydrogen production

## Abstract

A sustainable and effective method for de-oxygenation of few-layer graphene oxide (FLGO) by glycerol gasification in supercritical water (SCW) is described. In this manner, reduction of FLGO and valorization of glycerol, in turn catalyzed by FLGO, are achieved simultaneously. The addition of glycerol enhanced FLGO oxygen removal by up to 59% due to the in situ hydrogen generation as compared to the use of SCW only. Physicochemical characterization of the reduced FLGO (rFLGO) showed a high restoration of the sp^2^-conjugated carbon network. FLGO sheets with a starting C/O ratio of 2.5 are reduced by SCW gasification of glycerol to rFLGO with a C/O ratio of 28.2, above those reported for hydrazine-based methods. Additionally, simultaneous glycerol gasification resulted in the concurrent production of H_2_, CO, CH_4_ and valuable hydrocarbons such as alkylated and non-alkylated long chain hydrocarbon (C12–C31), polycyclic aromatic hydrocarbons (PAH), and phthalate, phenol, cresol and furan based compounds.

## 1. Introduction

Research on graphene-based materials has grown exponentially in the decade since Geim and Novoselov isolated and characterized a single layer of graphene in 2004 [1]. Graphene’s simple structure, composed of a two-dimensional conjugated network of sp^2^-hybridized carbon atoms, as well as its outstanding electronic, optical, thermal, mechanical and chemical properties [2,3,4], make it a leading material in diverse fields such as electronic devices, supercapacitors, batteries, solar cells, biological engineering, filtration, composites, catalysts, flexible transparent displays and sensors [5,6,7]. The main production methods to obtain graphene include top-down strategies, such as exfoliation of graphite and related structures, and bottom-up ones, like epitaxial growth by CVD or organic synthesis [5].

Oxidative exfoliation methods are top-down chemical approaches based on conventional methods initially intended for the oxidation of graphite, i.e., Brodie [8], Staudenmaier [9] and Hummers [10], which use strong acids and oxidants to achieve graphene layer separation by intercalation of oxygenated groups [2,11]. These methods allow graphene oxide (GO) to be subsequently produced in a liquid-phase process, where the exfoliation of graphite oxide in suspended and isolated layers of GO takes place under certain mechanical processes, enabling its mass production [2,5]. The production procedure is versatile, with a wide variety of graphitic precursors available, including carbon nanotubes, as well as numerous subsequent reduction methods to obtain reduced graphene oxide (RGO) [12,13,14]. Reduction methods recover the sp^2^-conjugated graphene network disrupted by oxygenated functionalities, such as hydroxyl and epoxide groups in basal planes and carboxylic and carbonyl groups on the edges [15,16,17], as a result of the previous oxidation step. However, none of the reported reduction methods completely remove the total anchored oxygen. Briefly, thermal annealing [12,18] and chemical [11,14] GO reduction approaches have been two traditional strategies, although photoreduction methods have also been explored [19]. Chemical reduction methods have the advantage of being carried out in liquid phase, which facilitates graphene functionalization. A large number of chemical reactants for liquid phase reduction are collected in recent specialized reviews [14,20]. However, the most effective chemical reduction processes in the literature involve toxic, explosive and aggressive chemical reducing agents such as hydrazine, hydroquinone or sodium borohydride, which hinder RGO mass production and processability. For this reason, a number of alternative approaches and green reducing agents have arisen, including vitamins, saccharides, amino acids, microorganisms, proteins and peptides, and plant extracts [20,21]. Common disadvantages such as cost, low reducing efficiency and high reduction time are challenges that green reduction agents must still overcome. Eco-friendly alternative reductive techniques such as solvothermal [22,23,24,25,26], hydrothermal [25,26,27,28,29,30,31,32,33], and electrochemical [34] reduction methods have been developed in parallel. Impurity-free RGO, simple setup, mild reduction conditions and controllable final degree of reduction are the main advantages of solvothermal/hydrothermal routes [21].

Hydrothermal methods, employing reductant-free superheated water at low temperatures (160–220 °C), have achieved a partial removal of oxygenated groups, reaching a C/O atomic ratio reduction from 2–4 in the starting GO [13] to 5.3–5.8 [29,32,33] but remaining below those reported for hydrazine (10.3–11.5) [11,14]. According to Zhou et al., [27] superheated water is more effective than hydrazine in restoring the conjugated sp^2^ network. In fact, the removal of oxygenated groups can be improved by increasing water pressure and temperature to near- or supercritical conditions (374 °C and 22.1 MPa). At supercritical conditions, water density, ionic product and dielectric constant are reduced while its diffusivity increase, which make it a highly reactive and homogeneous medium for reactions of heterolytic (ionic) bond cleavage [35]. Supercritical water (SCW) [36,37,38] and other supercritical fluids (SCF) such as alcohols [39,40,41,42,43], *N*,*N*-dimethylformamide (DMF) [39], *N*-methyl-pyrrolidone (NMP) [39], or CO_2_ [41,44], have been proven more effective in the de-oxygenation of GO than superheated fluids. Mungse et al. [36] observed that the degree of de-oxygenation measured by FTIR and XPS increased with temperature and hydrothermal pressure. They found that most functionalities were eliminated under supercritical conditions (above 380 °C), even ether (such as pyran, furan or pyrone) and phenol groups, which were thermally stable under superheated water below 300 °C.

In this communication, an unexplored sustainable approach for GO reduction via supercritical water gasification (SCWG) of glycerol is reported by the first time. Glycerol is a valuable green compound generated as a by-product of the biodiesel production process (at the ratio of 1 ton per 9 tons of biodiesel), which presents low toxicity, very low price, large availability and renewability [45]. The production of hydrogen, syngas or high-value intermediates as acrolein from SCWG of glycerol has been reported in literature in both catalytic and non-catalytic processes [46,47,48], working in a wide range of operating temperatures (300–850 °C) and pressures (23–45 MPa). Non-catalytic routes require temperatures above 650 °C and longer residence times for complete gasification of glycerol [49,50,51,52,53,54,55,56]. As for the catalytic process, homogeneous catalysts [53,54,57,58,59,60,61,62] such as Na_2_CO_3_, H_2_SO_4_, K_3_PO_4_, K_2_CO_3_, KOH or NaOH have been used to improve the carbon to gas conversion and the yield to hydrogen by means of the water-gas shift reaction (WGS; CO + H_2_O ↔ CO_2_ + H_2_). Heterogeneous catalysts based on transition metals such as Ni, Ru, Pt, Co, Cu or Zn [51,52,63,64,65,66,67,68,69,70,71,72] as well as on activated carbon [49], offered high selectivity and recyclability.

In this work, the sustainable reduction of few-layer graphene oxide sheets (FLGO) with a starting C/O ratio of 2.5 is studied by using glycerol in SCW at 400 and 500 °C. In this manner, reduction of FLGO and valorization of glycerol, in turn catalyzed by FLGO, are addressed simultaneously.

## 2. Results and Discussion

### 2.1. Reduced Graphene Oxide Characterization

**Figure 1:**
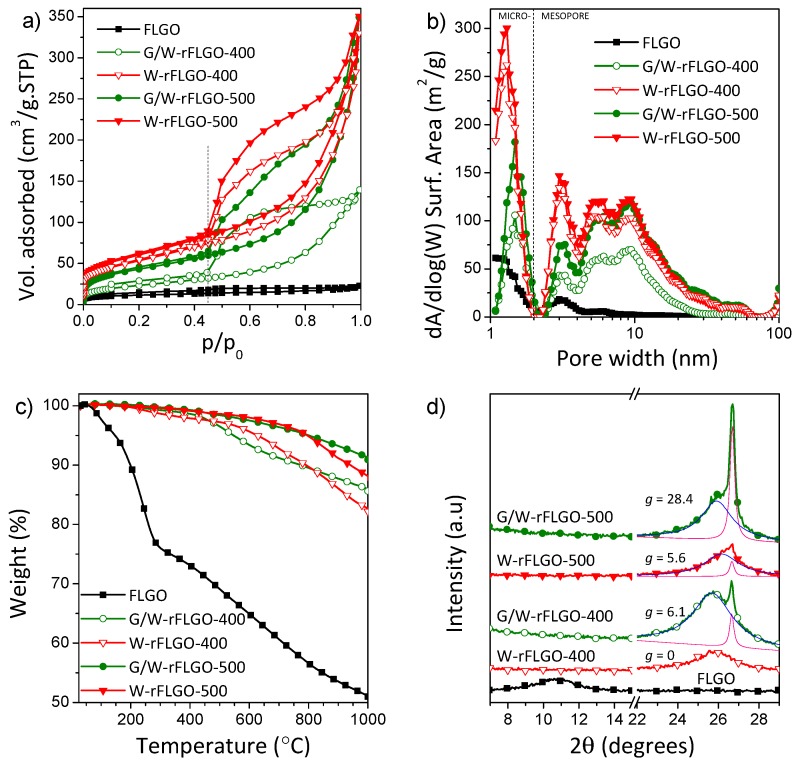
(**a**) Nitrogen adsorption-desorption isotherms at 77 K; (**b**) non-local-density functional theory (NLDFT) pore size distributions of FLGO and rFLGO; (**c**) thermal behavior of FLGO and rFLGO under N_2_ with a ramp rate of 5 °C min^−1^; (**d**) XRD patterns of FLGO and rFLGO.

The exposure of FLGO to SCW and SCWG of glycerol resulted in significant changes in its texture, thermal behavior and structure as revealed by Brunauer-Emmett-Teller (BET) analysis based on N_2_ adsorption, thermogravimetric analysis (TGA) and X-ray diffraction (XRD), respectively (Figure 1).

Based on the results of N_2_ physisorption shown in Figure 1a, FLGO and rFLGO exhibited type IV isotherms according to the International Union of Pure and Applied Chemistry (IUPAC) classification [73], typical of mesoporous solids, with a hysteresis loop that closed at relative pressures of p/p_0_ = 0.45, and evolved from H2 type in the case of FLGO to a combination of H2 and H3 types for reduced samples; both correspond to complex pore structures in which network effects are important. The drop after a saturation plateau in the desorption branch at p/p_0_ = 0.55–0.6 was greater in the case of H2 type hysteresis, and it is attributed to pore blocking in pore necks [73]. Furthermore, the type H3 hysteresis is given to non-rigid aggregates of plate-like particles, where condensation takes place in capillary spaces between parallel plates or open slit-shaped capillaries [73]. The BET surface area of FLGO increased after reduction treatments (see Table 1). Moreover, BET surface area increased more at 500 °C than during the equivalent processing at 400 °C. Reduction provides access for N_2_ to a clean graphene surfaces due to oxygen groups removal. rFLGO samples showed an inner interlayer stacking more accessible to gas than starting FLGO where oxygenated functional groups cause blockage of the pores. In FLGO, microporosity (26% of the BET surface area) and narrow mesoporosity (<5 nm) are mainly ascribed to the accessible cuneiform pores between the graphene layers (slit type pores). On the other hand, G/W-rFLGO samples presented a remarkable increase in surface area, but lower than that achieved with SCW only. As will be further discussed, the largest reduction of GO by glycerol gasification produced higher graphene layer restacking and folding of the starting FLGO with consequent loss of porosity. Figure 1b shows the pore size distributions from the adsorption branch analyzed by NLDFT. Supercritical reduction treatments developed the micro (1–2 nm) and mesoporosity of the FLGO. This effect was more pronounced in the case of W-rFLGO samples, which achieved above 42 m^2^ g^−1^ and 0.5 cm^3^ g^−1^ of micropore surface area and total pore volume, respectively, compared to 11 m^2^ g^−1^ and 0.035 cm^3^ g^−1^ in the starting FLGO (see Table 1). In addition, slightly narrower micropores were found for W-rFLGO (~1.3 nm) compared with G/W-rFLGO (~1.5 nm). Wide mesopores (5–60 nm) are attributed to structural defects, sheet overlapping and aggregate formation. These are responsible for the difference between the measured surface areas and the theoretical one for completely exfoliated and isolated graphene sheets (~2620 m^2^ g^−1^) [74].

The thermal stability of the samples was analyzed by TGA. Figure 1c shows the weight loss as a function of temperature for FLGO and rFLGO samples at a heating rate of 5 °C min^−1^ under N_2_ atmosphere. A 25 wt. % weight loss was registered before 300 °C for FLGO, and it is attributed to the release of water molecules intercalated between graphene oxide layers (up to 100 °C) and the evolution of labile oxygenated groups to CO and CO_2_ from 150 °C onwards [11]. After 300 °C, the removal of more stable oxygen functionalities takes place. The weight loss in rFLGO is minimal before 400 °C, indicating that water molecules and most of the oxygen-containing functional groups were removed after the supercritical reduction process both in presence and absence of glycerol. G/W-rFLGO showed a slightly higher temperature resistance achieving residual carbon fractions (residue at 1000 °C) of 86.0 and 91.3% for 400 and 500 °C temperature reduction, respectively, compared to 82.7 and 88.1% of W-rFLGO, due to a greater graphitic restoration and de-oxygenation, with increasing Van der Waals forces between layers [75].

XRD patterns of the FLGO compared to those of the rFLGO are depicted in Figure 1d. As a result of the oxidative treatment and ultrasound exfoliation of multi-wall carbon nanotubes (MWCNT), the intercalation of oxygenated groups generates FLGO sheets with well-separated graphitic layers. The diffraction peak of the basal plane (002) of graphite stacking (centered at a scattering angle (2θ) of 26.5° in MWCNT; *d*-spacing = *d*_002_ = 0.336 nm) shifted to 10.7° in FLGO, corresponds to a *d*-spacing of 0.830 nm commonly attributed to GO. Supercritical reduction recovered the (002) peak at 25.7–26.3° at the expense of shifted (002) driven by the approach of graphene layers after the removal of intercalated oxygenated groups. The contribution of two peaks is observed in some XRD patterns (Lorentzian fitting deconvolution has been included in Figure 1d), being this fact more pronounced in the case of G/W-rFLGO. These peaks correspond to two types of crystalline structures: disordered or turbostratic graphite (at 25.7–26.3°) and hexagonal graphite (at 26.7°) with different stacking ordering degrees and *d*-spacing: 0.340–0.346 and 0.334 nm, respectively [76]. This strongly suggests the coexistence of corrugated or disordered FLGO sheets and graphitic stacking regions [77]. The graphitization degree, *g* (included in Figure 1d), was measured as a ratio of the integrated areas of the turbostratic and graphitic contributions (*g* = (*A*_graphite_/(*A*_turbostratic_ *+ A*_graphite_)) × 100%) [78]. As can be observed, the graphitization degree was higher in G/W-rFLGO samples, reaching 28.4% at 500 °C. The severity of the treatment by SCWG of glycerol resulted in the formation of graphite domains by restacking of graphene layers due to both strong π-π interactions and Van der Waals forces between graphene basal planes and intercalated water molecules [11,79,80,81]. This larger restacking in the G/W-rFLGO samples resulted in smaller BET surface areas as previously seen by N_2_ physisorption (see Table 1). The reduction caused in all cases an increase in the number of graphene layers (*L_c_*/*d*_002_ + 1) from 4 in starting FLGO (*L_c_* = 3.1 nm) to ca. 10 layers in rFLGO (*L_c_* = 3.4–4.4 nm).

**Figure 2:**
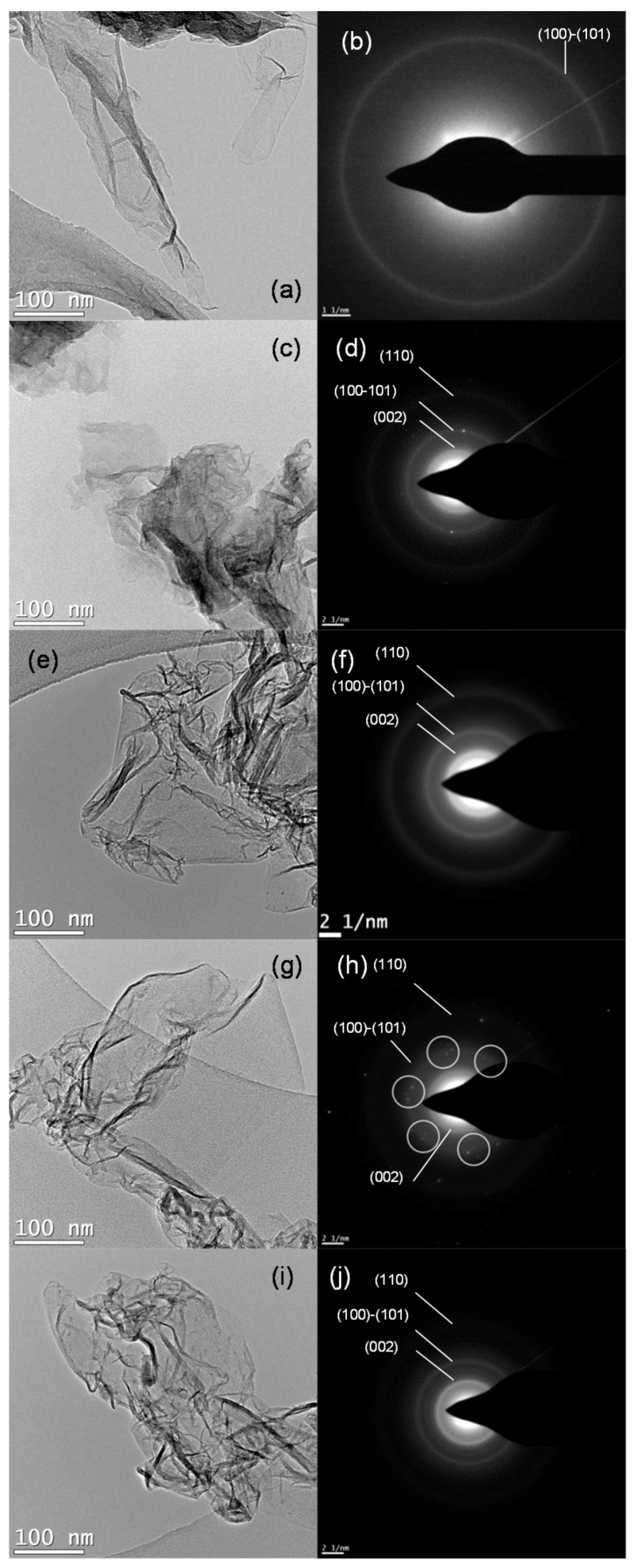
TEM images and SAED patterns of (**a**,**b**) FLGO sheets; (**c**,**d**) G/W-rFLGO-400; (**e**,**f**) W-rFLGO-400; (**g**,**h**) G/W-rFLGO-500 and (**i**,**j**) W-rFLGO-500.

TEM images in Figure 2 reveal the transparent and two-dimensional morphology of FLGO and rFLGO samples, showing micrometric folding structures. The 2D structure of graphene is thermodynamically stable via bending and wrinkling [82]. Consequently, FLGO and rFLGO presented corrugated and scrolled structures. However, FLGO had flatter and bumpy sheets due to the large amounts of hydroxyl and epoxide groups in graphene basal planes [18]. As the spine of a book, multilayer graphitic staking previously identified in the XRD deconvolution, can be visualized as the backbone of the emerging wrinkled sheets of graphene. In Figure S1 in the suporting information some HRTEM images of folds and multilayer graphitic type stacking can be observed. As a result of the reduction process, rFLGO samples present a shortening of the FLGO sheets and a better definition of folds and edges. Although the glycerol-assisted reduction (G/W-rFLGO) results in a smaller modification of the original appearance of the FLGO sheets, the most important difference can be found in the temperature used in the treatment of reduction: the higher the temperature, the greater the agglomeration of these leaves (additional TEM images of FLGO and rFLGO can be found in Figure S2 in the Supplementary Material). The selected area electron diffraction (SAED) patterns accompanying Figure 2 show ring-patterns formed by spot merging and accumulation, typical of polycrystalline samples with randomly oriented grains [83]. Distinguishable bright spots corresponding to the sixfold symmetry of stacking in graphite [84] are located over G/W-rFLGO diffraction rings (marked in Figure 2h), which indicates a higher sp^2^ graphene network restoration.

**Figure 3:**
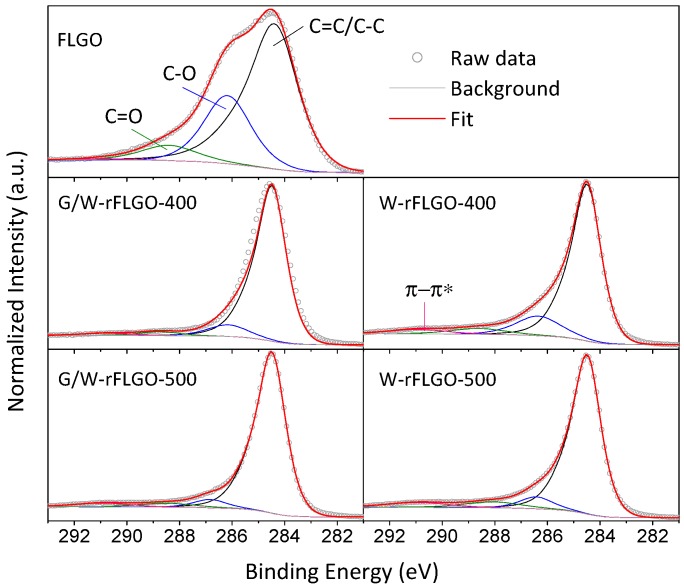
Deconvoluted C1s peak of XPS spectra of FLGO and rFLGO sheets.

X-ray photoelectron and energy-dispersive X-ray spectroscopies (XPS and EDX, respectively) were used to quantify the atomic composition of FLGO and rFLGO samples. Three components were deconvoluted by Gaussian/Lorentzian (40:60) fitting from XPS C1s high-resolution spectra as shown in Figure 3: sp^2^ graphitic carbon (C=C) and sp^3^ hybridized carbon (C–C) are combined in an asymmetric peak at 284.5 eV [85], C–O bonds in hydroxyls (C–OH) or epoxides (C–O–C) at 286.1–286.8 eV, and C=O bonds in carbonyls (C=O) or carboxyl (O=C-OH) at 288.1–289.0 eV. An additional peak at 290.9 eV attributed to the π-π* shake up satellite contribution was found for rFLGO. The atomic composition of FLGO and rFLGO calculated from both deconvoluted XPS C1s spectra and EDX analyses are summarized in Table 2. According to XPS results, FLGO, which had an initial oxygen content of 30.3 at. % (C/O ~ 2.3), was effectively reduced by removal of oxygen-containing functional groups resulting in oxygen contents in rFLGO ranging from 14.5 at. % to 8.1 at. %. Moreover, the reduction of FLGO was consequently accompanied by sp^2^-carbon recovery and the inherent appearance of the π-π* transition. Consequently, there were remarkable increases of the C/O ratio for W-rFLGO and G/W-rFLGO. In addition, SCW treatment and to an even further extent glycerol SCWG removed both the in-plane oxygenated groups (hydroxyls and epoxides) and out-plane groups (carbonyls and carboxyls). Focusing on the temperature effect, at 500 °C higher C/O ratios were achieved with and without glycerol. Contrasting now the composition values obtained by EDX, oxygen contents were somewhat lower than those calculated by XPS, since the latter is a surface technique and oxygen is more abundant on the surface of the graphene/graphite stacks. The difference in oxygen content is greater when there is an excessive restacking of the reduced graphene-oxide layers (as observed for G/W-rFLGO-500 in Figure 1d); hence G/W-rFLGO-500 showed a greater difference in oxygen content as measured by EDX, resulting in a bulk C/O ratio of 28.2, much higher than that calculated by XPS. Note that this measurement problem observed for the XPS technique has not been previously reported since the C/O ratios found in literature correspond to RGO whose starting material is not FLGO and therefore does not present restacking after hydro- or solvothermal reduction. The addition of glycerol enhanced FLGO oxygen removal by up to 59% as compared to the use of SCW only. In this case, FLGO simultaneous reduction with glycerol SCWG achieved greater oxygen-containing group removal and sp^2^-conjugated graphene network restoring, as shown by TGA, XRD, SAED, EDX and XPS analysis, than reduction in SCW method and the previously reported green or hydrothermal-based reduction methods. As shown below, this reflects the effectiveness of H_2_ produced in situ from glycerol gasification to react with the oxygenated groups in FLGO.

### 2.2. Products of the Supercritical Water Gasification of Glycerol

**Figure 4:**
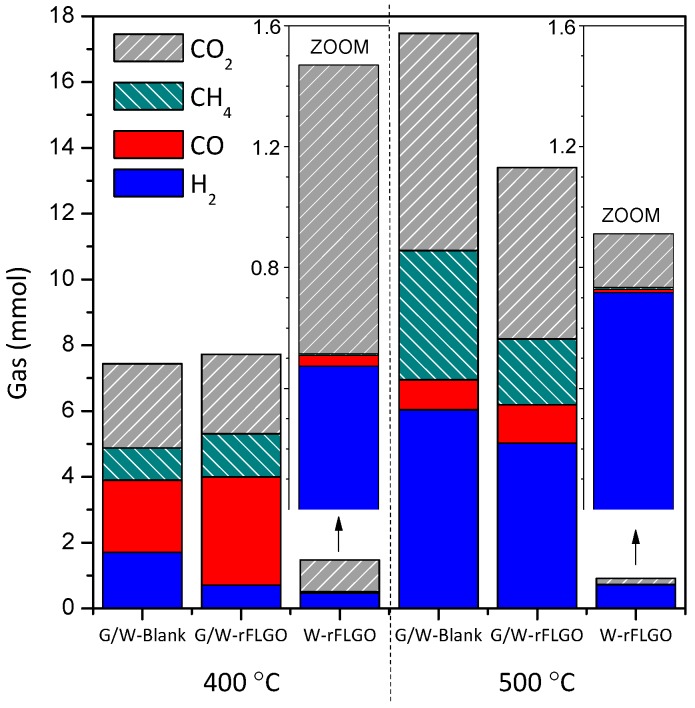
Gas recovered after FLGO reduction by means of SCW (W-rFLGO samples) and SCWG of glycerol (G/W-rFLGO samples) at 400 and 500 °C. Blanks (SCWG of glycerol without FLGO) were included for both temperatures. Zoom of W-rFLGO were included in order to facilitate the comparison.

Gas composition after reaction was analyzed by gas chromatography to evaluate the gas obtained in reduction of FLGO in water or glycerol/water under supercritical conditions, as shown in Figure 4 (composition, molar quantities, gas yields and carbon-to-gas efficiency data are collected in Table S1 in the Supplementary Material). The gas fraction was mainly composed of H_2_, CO, CO_2_ and CH_4_ in varying proportions. As expected, higher gas formation was obtained at higher temperature (500 °C), due to the increased production of H_2_, CO_2_ and CH_4_. On the contrary, CO formation decreased at 500 °C. Increased gas production was originated by SCWG of glycerol. The product distribution was also determined by the WGS reaction, which leads to additional H_2_ and CO_2_ by CO consumption [57]. In addition, higher temperatures favor free-radical reactions resulting also in higher gas formation [50,57]. CH_4_, in turn, is produced by pyrolysis of glycerol intermediates or by methanation reactions (CO + 3H_2_ ↔ CH_4_ + H_2_O) [57]. Heterogeneous catalysts were demonstrated to favor the carbon to gas efficiency and the WGS reaction [52,65,66,67,69], which in this case would displace even further towards the formation of H_2_ and CO_2_ due to H_2_ consumption in the reduction of the FLGO. On the contrary, the methanation reaction towards the formation of CH_4_ would not be favored initially. Glycerol gasification (and hence hydrogen production) could be improved by using shorter residence times, lower concentrations of glycerol in the feed and/or higher operating temperatures [51,66,70], but could nevertheless cause gasification of FLGO. Therefore, the optimum experimental conditions used to GO reduction should be carefully selected, and this point should be addressed in future works.

In tests with SCW only (leading to W-rFLGO), gas formation was much lower than when glycerol was used and gas products were mainly H_2_ and CO_2_ as a result of FLGO gasification and evolution of oxygen-containing functional groups in FLGO [86,87]. Low amounts of CO and CH_4_ were also measured, latter indicates that methanation would not be favored under these conditions. Note that SCW in the absence of FLGO did not produce gas. Hence, H_2_ produced from glycerol and, presumably, the self-generated overpressure would improve FLGO reduction, contributing to the differences between G/W-rFLGO and W-rFLGO observed in the physicochemical characterization and C/O ratios obtained.

It has been previously reported that pressure and H_2_ atmosphere enhanced the GO reduction [33]. H_2_ consumption in FLGO reduction simultaneous with SCWG of glycerol was ca. 0.41 mmol per mmol^−1^ of oxygen initially present in of FLGO at both temperatures (calculated by difference between the H_2_ productions of G/W-rFLGO and its blank), and therefore there was a H_2_ surplus, especially at 500 °C. This surplus could potentially be used to develop a process that couples FLGO reduction with hydrogen production, which would imply a further improvement towards larger scale operation in a continuous reactor than reduction in SCW alone, although residence times should be shortened in order to make the operation viable. In addition to the gases from glycerol and FLGO gasification, liquid products were collected (changes in appearance can be seen in the photographs attached in the Figure S3 in the Supplementary Material) and analyzed by chromatography. Liquids are classified by groups and listed in Table S2 in the Supplementary Material. Due to an amount two orders of magnitude lower (by weight), liquids products of FLGO reduction in SCW (without glycerol; samples: W-rFLGO-400 and W-rFLGO-500) could not be measured. SCWG of glycerol (reactions with or without FGLO) promoted the aromatization and polycondensation of glycerol into alkylated and non-alkylated long chain hydrocarbons (C12–C31), polycyclic aromatic hydrocarbons (PAH) and phthalates. Aromatization of glycerol was reported for acid catalysts as zeolites, which is known to form alkylaromatics (mainly C8–C10) [88], including benzene and alkylbenzenes (mainly toluene, xylene and trimethylbenzenes) [89] from glycerol. All these products appeared in greater quantities in the presence of FLGO, which also led to the formation of phenol, cresol and furan-based compounds. The formation of linear carbon chains from graphene was earlier reported by Iijima et al. [90], but in this case the FLGO introduced into the system is thought to catalyze their formation from glycerol in SCW as an increase in their concentrations was observed respect to the blank. However, the quantification and formation mechanism of these products need to be further investigated. In summary, the high reduction effectiveness combined with the use of a valuable and green chemical like glycerol makes this a very interesting method for GO reduction and the simultaneous production of hydrogen, syngas and other valuable hydrocarbons.

## 3. Materials and Methods

### 3.1. Graphene Oxide Obtention by Multi-Wall Carbon Nanotubes Unzipping

A suspension of FLGO sheets was obtained by unzipping of MWCNT previously produced by catalytic decomposition of methane in a rotary bed reactor [91,92]. Briefly, MWCNT were obtained in 180 min runs with a feed of pure methane (99.99%) at 750 °C, with a weight hourly space velocity of 1.5 l_N_·g_cat_^−1^·h^−1^, and using a Fe-Mo/MgO catalyst (molar composition: 63.2:5.1:31.6) [91,92]. Prior to FLGO obtention, MWCNT were purified by acid treatment with concentrated HNO_3_ during 30 min at boiling temperature to eliminate metal particles (from the remaining catalyst) and generate oxygenated surface groups, which have a relevant importance in its subsequent unzipping [93]. These carbon materials exhibited similar structural properties to those of graphite, with an interlayer spacing of 0.3367 nm, and a surface area of 45.3 m^2^·g^−1^. In Figure S4 in the supponting information some representative TEM images of the purified MWCNT can be observed. The unzipping procedure was based on the modified Hummers method [2,10,92,93,94] followed by ultrasonic exfoliation. For FLGO obtention, 1.5 g of MWCNT, 1.5 g of NaNO_3_ (purity ≥ 99.0%), and 69 mL of H_2_SO_4_ (96%) were stirred together in an ice bath. 13.8 g of KMnO_4_ was slowly added to the mixture under vigorous stirring to obtain a ratio of KMnO_4_ to as prepared MWCNT according to the optimum oxidation ratio (OR) obtained in a previous work OR = KMnO_4_/MWCNT = 10 (wt. %; excluding the catalyst content) [92]. After addition of KMnO_4_, temperature was kept below 20 °C during mixing and then the solution was stirred at 30 ± 5 °C for 2 h and at room temperature overnight. After oxidation, a brownish thick paste was formed. Then, 120 mL of deionized water was slowly added while maintaining the temperature below 70 °C. Subsequently, the solution was stirred for 60 min and was diluted with 300 mL of deionized water. A volume of 14 mL of H_2_O_2_ (33%) was added dropwise, turning the solution colour to yellowish brown. Material exfoliation was carried out by sonication for 4 h. The product was washed by centrifugation at 9500 rpm with HCl (10%) and deionized water until neutral pH. GO-based suspension with a concentration of 13.2 mg/mL was separated from unreacted MWCNT and coarse particles as supernatant at 1000 rpm and cycles of 10 min. Finally, FLGO were dried at 65 °C overnight. After the oxidation/exfoliation method FLGO powder was completely free of impurities (including the catalyst residues from the MWCNT synthesis), as confirmed by inductively coupled plasma-optical emission spectrometry (ICP-OES) (not shown).

### 3.2. Reduction of FLGO by Supercritical Water Gasification of Glycerol

For the reduction method presented here, FLGO sheets from MWCNT unzipping were used; however, this reduction method may also be applied for any graphene oxide material, including those obtained by the conventional graphite oxidation process. To perform reduction reactions a stainless steel microbomb batch reactor was used (a detailed diagram is shown in the Figure S5 in the Supplementary Material) [95]. The reactor consists of two sections, a reaction section and a gas recovery section, isolated one from the other with a heavy duty high T and high P needle valve. The reaction section has a volume of 12 mL and is comprised of a 1/2 inch Swagelok bored-through tee with two ends plugged. The tee is connected by means of a 1/4 inch tube to the high T and high P needle valve. The gas product recovery section is equipped with a pressure gauge to determine the pressure of the gas produced after reaction and with an integrated septum that enables the sampling of gas for analysis.

In a typical run, 0.130 g of dried FLGO were dispersed in a proper volume of glycerol/water solution (18 wt. %; glycerol purity > 99%, GC grade, Sigma-Aldrich, London, UK) and placed in the stainless steel tee before attaching it to the rest of the reactor. The reactor was purged with He and closed at ambient pressure to ensure an oxygen free atmosphere to start the experiment. Volumes of 4 and 2.9 mL of glycerol/water solution guaranteed supercritical water conditions during reaction at 400 and 500 °C, respectively, resulting in a pressure of 230 bar. The corresponding water pressure in the system was calculated using data from National Institute of Standards and Technology (NIST) [96]. During operation the reactor was placed inside a heated fluidized sand bath and connected to a reactor shaker assembly for agitation. Reactions were carried out at 400 or 500 °C and a reaction time of 2 h, defined as the holding time at the desired reaction temperature. At the end of the run, the reactor was quenched with cold water to ambient temperature to stop reduction and then depressurized. rFLGO produced from these reactions at 400 and 500 °C were denoted as “G/W-rFLGO-400” and “G/W-rFLGO-500”, respectively. Additional runs in SCW without glycerol (“W-rFLGO-400” and “W-rFLGO-500”) and blanks (glycerol/water without FLGO: “G/W-Blank-400” and “G/W-Blank-500”) were performed to determine the effect of glicerol. All sample identifications and conditions are summarized in Table S3 in the Supplementary Material. Gas products were collected and analyzed through gas chromatography while solid and liquid products were carefully recovered from the reactor with a solvent mixture of CHCl_3_/H_2_O 1:1 *v*/*v*. rFLGO were separated from the liquid products by centrifugation at 3800 rpm and then dried at room temperature under an inert gas flow (N_2_ oxygen free). After solvent removal by evaporation in N_2_ at room pressure and temperature, liquids were analyzed by gas chromatography-mass spectrometry (GC-MS).

### 3.3. Characterization Techniques

X-ray diffraction (XRD) of FLGO and rFLGO were acquired in a Bruker D8 Advance Series 2 diffractometer (Billerica, MA, USA). The angle range scanned was 4–55° using a counting step of 0.02° and a counting time per step of 4 s. XRD data were fitted using the structure analysis software TOPAS (Bruker, Billerica, MA, USA) including subtraction of a Chebyshev polynomial background and peak deconvolution by fitting to a split pseudo-Voigt functions. The graphite peak fit gave information on structural parameters such as interlayer spacing (or *d*-spacing), *d*_002_, or mean crystallite size along *c* axis (transverse to the graphene planes), *L_c_*. The mean interlayer spacing was evaluated from the position of the (002) peak applying Bragg’s Law [97], while the mean crystallite size was calculated using the Scherrer formula, with a values of *K* = 0.89 [97]. From these, the average number of graphene layers, *n*, was calculated as (*L_c_*/*d*_002_) + 1 [92].

X-ray photoelectron spectroscopy (XPS) analyses were carried out with an ESCAPlus OMICROM system equipped with a hemispherical electron energy analyser. The spectrometer was operated at 15 kV and 15 mA (225 W), using a non-monochromatic MgKα X-ray source (*hv* = 1253.6 eV) and under vacuum (<5 × 10^−9^ Torr). Adventitious carbon is removed by argon sputtering (200 eV, 25 mA, 10 min). Analyser pass energy of 50 eV was used for survey scans and 20 eV for scans of C1s region. For calibration purposes, the C1s binding energy of the graphitic peak (BE) was referenced at 284.5 eV. A survey scan (1 sweep/200 ms dwell) was acquired between 1200 eV and 0 eV. The CASA XPS data processing software allowed smoothing, Shirley type background subtraction, peak fitting and quantification.

The textural properties of the FLGO and rFLGO were measured by N_2_ adsorption at 77 °C in a Micromeritics ASAP2020 apparatus. The specific surface areas and total pore volumes were calculated by applying the BET method to the respective N_2_ adsorption isotherms and the pore size distributions from the adsorption branch analyzed by NLDFT.

Morphological appearance of FLGO and rFLGO were performed by transmission electron microscopy (TEM) using a Tecnai F30 (FEI) microscope, equipped with a cannon of 300 KeV and a maximum resolution of 1.5 Å. In addition, an energy dispersive X-ray (EDX) analyzer Röntec XFlash Si(Li) coupled to a Hitachi S-3400N scanning electron microscope (SEM) was used to study the bulk composition of the samples.

Thermogravimetric analysis (TGA) was carried out in a NETZSCH TG 209 F1 Libra thermobalance (Selb, Germany). TGA profiles were obtained from a sample amount of 8 mg and using a heat rate of 5 °C min^−1^ within a temperature range from room temperature to 1000 °C under a flow rate of 50 mL min^−1^ of N_2_.

Gas chromatography with mass spectrometry analysis of the liquid product was performed in a Varian Star 3400/Saturn 2000 GC-MS equipped with a non-polar HT-5 (25 m × 0.32 mm) column from SGE (London, UK). The products were identified by comparison with the spectrum found in the instrument library. Gas products were analysed in a Perkin Elmer Clarus 500 gas chromatograph fitted with a thermal conductivity detector (TCD). The GC-TCD was equipped with a Carboxen 1010 plot capillary column (30 m × 0.53 mm).

## 4. Conclusions

A sustainable and effective method to reduce GO-based materials using simultaneous glycerol gasification in SCW was demonstrated. Glycerol is a byproduct of biodiesel production and can revalue through use in continuous processes as reduction reactions in supercritical conditions. In this manner, reduction of FLGO and valorization of glycerol have jointly been achieved, obtaining G/W-rFLGO by SCWG of glycerol at 500 °C with a C/O ratio of 28.2, well above those of the traditionally hazardous hydrazine-based methods (C/O ~ 10.3–11.5). In this way, it was demonstrated that the sp^2^-conjugated graphene network was restored through the removal of basal oxygenated groups. In addition, H_2_, CH_4_, CO_2_ and CO gases were recovered. The H_2_ produced by glycerol gasification enhanced the FLGO reduction in SCW, achieving greater oxygen removal than the recently reported process of reduction by SCW or SCF. Simultaneous glycerol gasification could be used to reduce FLGO with the concurrent production of H_2_, CO, CH_4_ and valuable hydrocarbons.

## Figures and Tables

**Table 1:** Textural parameters of FLGO and rFLGO.

	S_BET_ [m^2^ g^−1^] ^1^	S_mic_ [m^2^ g^−1^] ^2^	V_t_ [cm^3^ g^−1^] ^3^
FLGO	42	11	0.035
W-rFLGO-400	191	42	0.510
W-rFLGO-500	215	47	0.542
G/W-rFLGO-400	84	16	0.216
G/W-rFLGO-500	155	26	0.540

**^1^** BET surface area. ^2^ NLDFT micropore area. ^3^ Total pore volume at p/p_0_ = 0.996.

**Table 2:** XPS and EDX atomic compositions of FLGO and rFLGO.

	XPS Analysis [at. %] ^1^							EDX Analysis [at. %]		
	C=C/C–C	C–O	C=O	π-π*	C	O	C/O	C	O	C/O
FLGO	47.6	45.4	6.7	0.3	68.6	30.3	2.3	69.9	27.8	2.5
W-rFLGO-400	76.1	16.3	4.2	3.4	85.5	14.5	5.9	88.1	11.2	7.9
W-rFLGO-500	80.9	7.3	6.4	5.5	88.8	11.2	8.0	90.8	8.3	10.9
G/W-rFLGO-400	85.4	9.2	3.1	2.3	91.0	9.0	10.2	92.6	7.4	12.4
G/W-rFLGO-500	86.3	5.1	4.6	4.0	91.9	8.1	11.4	96.6	3.4	28.2

**^1^** Values obtained from C1s deconvolution.

## Supplementary Materials

The following are available online at http://www.mdpi.com/2079-4991/7/12/447/s1, Figure S1: HRTEM images of folds in some selected rFLGO samples, Figure S2: TEM images of (a,b) FLGO flakes; (c,d) G/W-rFLGO-400; (e,f) W-rFLGO-400; (g,h) G/W-rFLGO-500 and (i,j) W-rFLGO-500, Table S1: Compositions, molar quantities, gas yields and carbon-to-gas efficiencies of gas products, Figure S3: Photographs of liquid intermediates after solid removal by centrifugation (includes the extraction solvent: CHC_l3_/H_2_O), Table S2: Liquids intermediates from blanks and FLGO reduction by SCW and SCWG of glycerol, Figure S4: TEM images of purified MWCNT, Table S3: Sample identifications and conditions, Figure S5: Schematic of the micro-bomb batch reactor. (a) Purge inlet; (b) Gas pressure gauge; (c) Gas sampling port; (d) Purge outlet; (e) High pressure-temperature needle valve; (f) Type K thermocouple; (g) ½” in borethrough tee.
