# Supplementary material for: Enhanced Reduction of Few-Layer Graphene Oxide via Supercritical Water Gasification of Glycerol
Source: Nanomaterials (Basel). 2017 Dec 14;7(12):447. doi: 10.3390/nano7120447 (PMC5746937; doi:10.3390/nano7120447)
Supplement: Supplementary file 1 [file nanomaterials-07-00447-s001.pdf]

Supplementary Materials

Enhanced Reduction of Few-Layer Graphene Oxide via Supercritical Water Gasification of Glycerol

Daniel Torres [1], Pedro Arcelus-Arrillaga [2], Marcos Millan [2], José Luis Pinilla [1,*] and Isabel Suelves [1]

[1] Instituto de Carboquímica, CSIC, Miguel Luesma Castán 4, Zaragoza 50018, Spain; dtorres@icb.csic.es (D.T.); isuelves@icb.csic.es (I.S.)

[2] Department of Chemical Engineering, Imperial College London, London SW7 2AZ, UK; pedro.arcelus-arrillaga09@imperial.ac.uk (P.A.-A.); marcos.millan@imperial.ac.uk (M.M.)

* Correspondence: jlpinilla@icb.csic.es; Tel.: +34-976-733-977

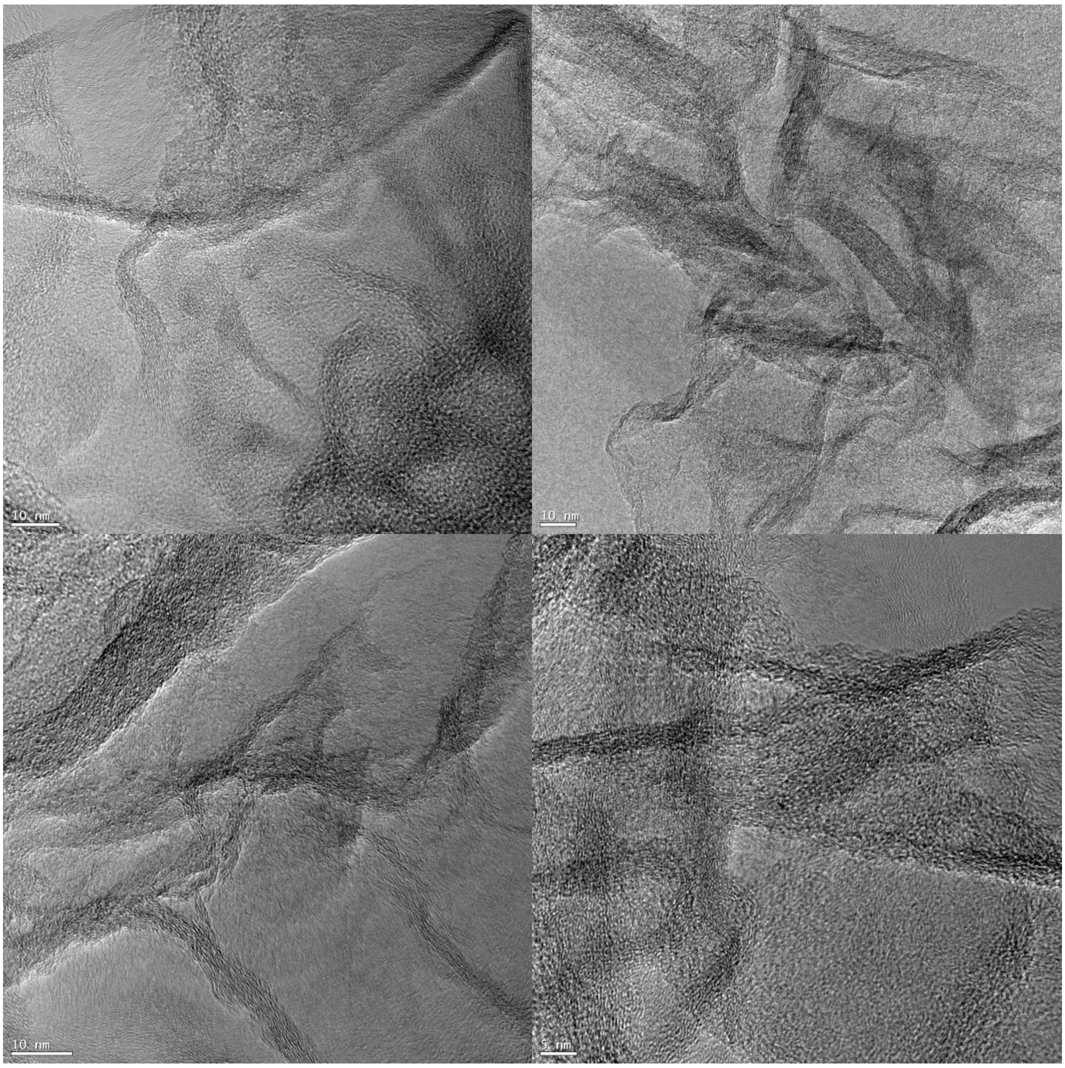

Figure S1. HRTEM images of folds in some selected rFLGO samples.

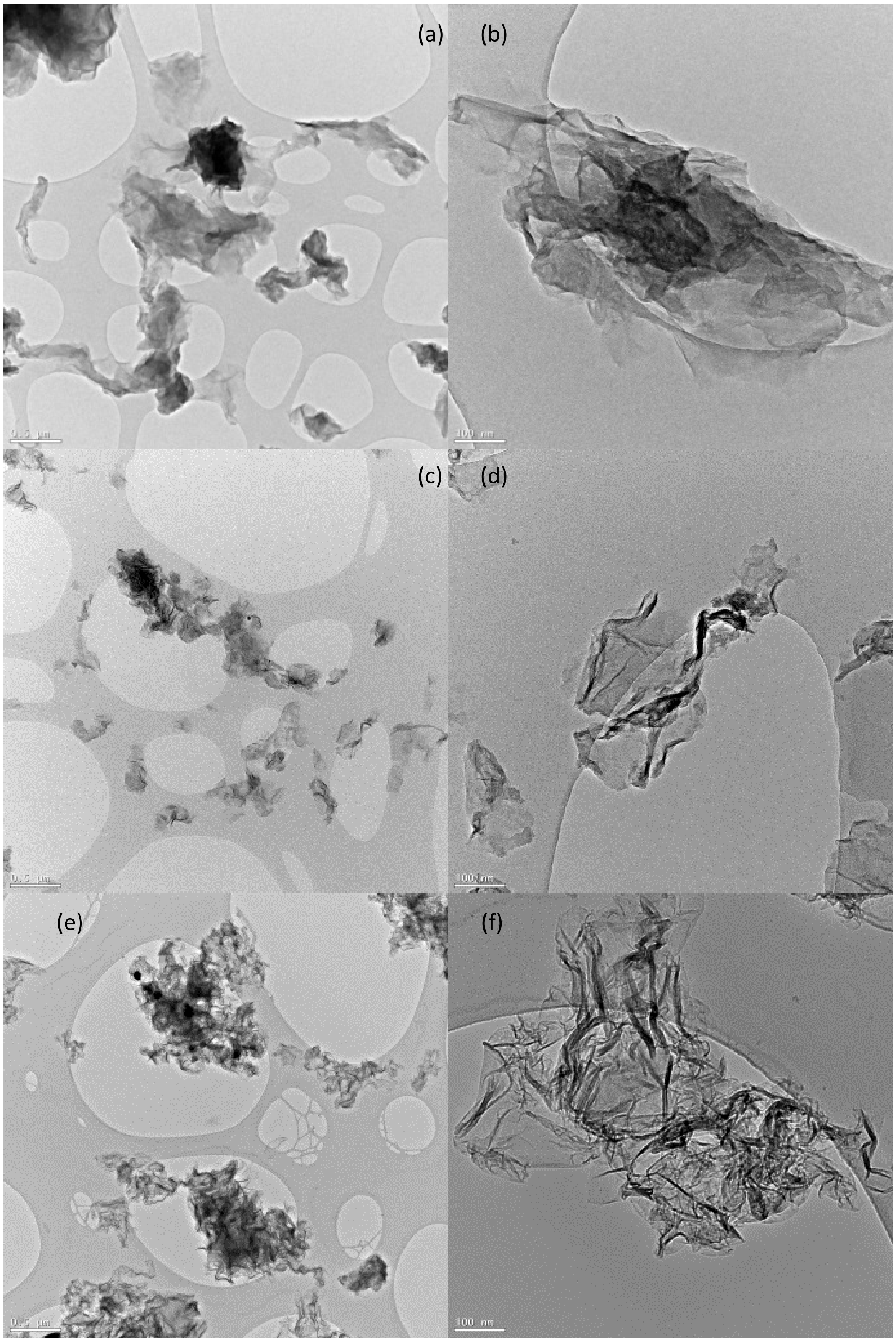
(a)
(b)
(c)
(d)
(e)
(f)
0.5 µm
100 nm

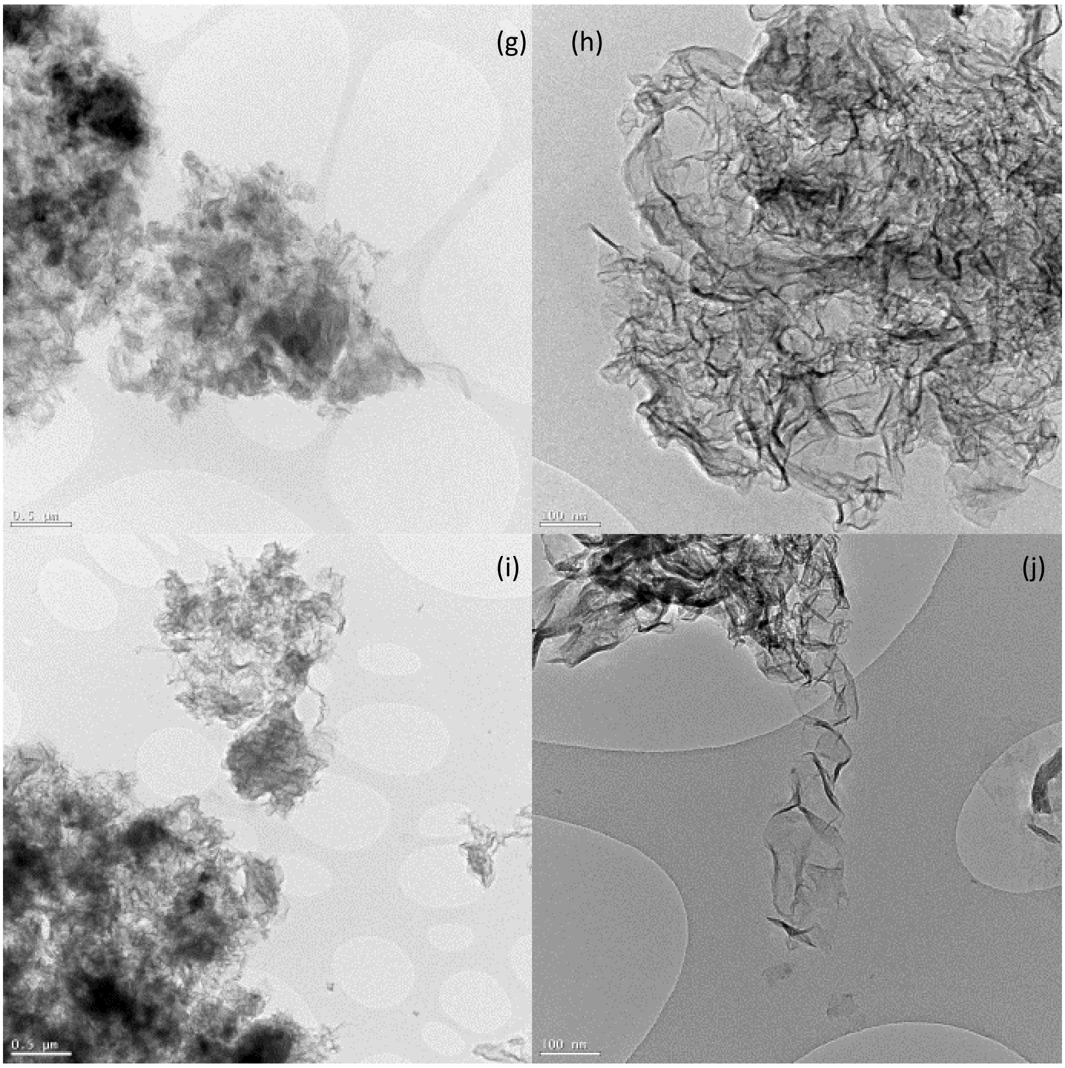

Figure S2. TEM images of (**a-b**) FLGO flakes; (**c-d**) G/W-rFLGO-400; (**e-f**) W-rFLGO-400; (**g-h**) G/W-rFLGO-500 and (**i-j**) W-rFLGO-500.

Table S1. Compositions, molar quantities, gas yields and carbon-to-gas efficiencies of gas products.

**Sample**	***n*** **[mmol]**	**Vol [%]**				***n*** **[mmol]**				**Gas yield [mmol mmol⁻¹ glycerol]**				**Carbon-to-gas efficiency* [%]**
		H_2	CO	CH_4	CO_2	H_2	CO	CH_4	CO_2	H_2	CO	CH_4	CO_2	
G/W-Blank-400	7.4	22.9	29.5	13.1	34.5	1.71	2.19	0.97	2.56	0.21	0.28	0.12	0.32	24.1
G/W-rFLGO-400	7.7	9.2	42.6	17.0	31.3	0.71	3.29	1.31	2.41	0.09	0.41	0.17	0.30	29.4
W-rFLGO-400	1.5	32.2	2.5	0.3	64.9	0.47	0.04	0.01	0.95	NG	NG	NG	NG	NG
G/W-Blank-500	17.5	34.6	5.2	22.5	37.7	6.04	0.91	3.93	6.60	1.03	0.15	0.67	1.12	64.8
G/W-rFLGO-500	13.4	37.5	8.7	15.0	38.8	5.02	1.16	2.01	5.21	0.85	0.20	0.34	0.88	47.4
W-rFLGO-500	0.9	78.6	1.2	0.8	19.5	0.72	0.01	0.01	0.18	NG	NG	NG	NG	NG

* calculated as $(n_{CO} + n_{CH_4} + n_{CO_2})/3n_{Glycerol}$ x 100; NG = non glycerol tests.

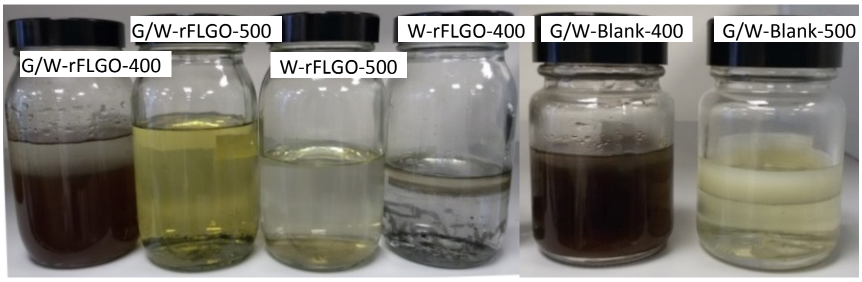

Figure S3. Photographs of liquid intermediates after solid removal by centrifugation (includes the extraction solvent: $CHCl_3/H_2O$).

Table S2. Liquids intermediates from blanks and FLGO reduction by SCWG of glycerol.

	G/W-Blank-400	G/W-Blank-500	G/W-rFLGO-400	G/W-rFLGO-500
Phenol and alkylphenols	-	-	✓✓	✓✓
Cresol/P-cresol	-	-	✓	✓
Furans	-	-	✓	✓
PAH (including naphthalene, alkylnaphthalene and polyaromatic compounds with 3 or more rings)	✓	✓	✓✓	✓✓✓
Phthalate (including alkylphthalates)	✓	✓	✓	✓
Long chain hydrocarbons (C7–C31, include alkylalcanes)	✓✓	✓✓	✓✓✓	✓✓✓

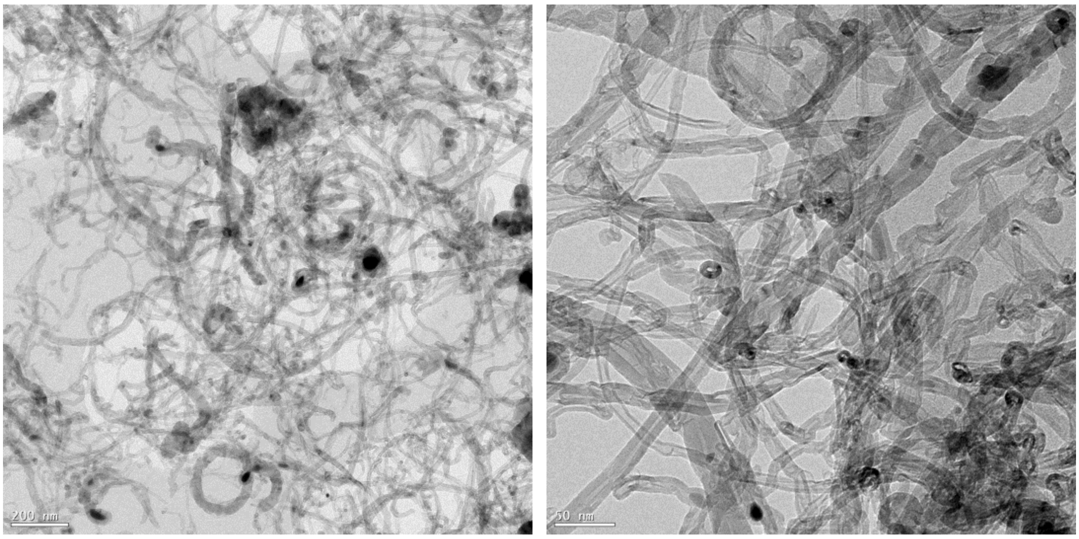

Figure S4. TEM images of purified MWCNT.

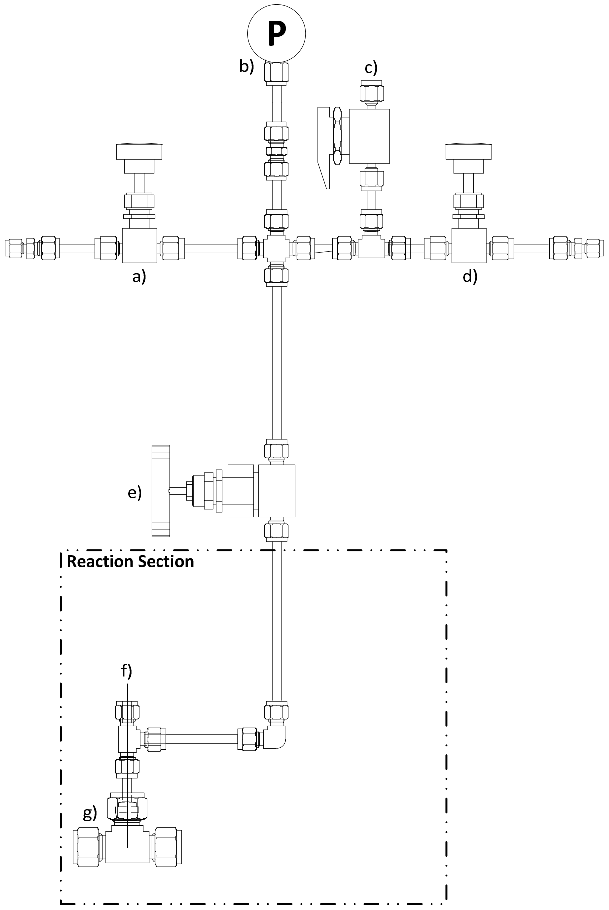

Figure S5. Schematic of the microbomb batch reactor. (**a**) Purge inlet, (**b**) Gas pressure gauge, (**c**) Gas sampling port, (**d**) Purge outlet, (**e**) High pressure-temperature needle valve, (**f**) Type K thermocouple, (**g**) ½″ borethrough tee.

Table S3. Sample identifications and conditions.

Sample ID	Glycerol	FLGO [mg]	T [°C]	Time [min]
G/W-Blank-400	YES	0	400	120
G/W-Blank-500	YES	0	500	120
G/W-rFLGO-400	YES	130	400	120
W-rFLGO-400	No	130	400	120
G/W-rFLGO-500	YES	130	500	120
W-rFLGO-500	No	130	500	120
